# Pharmacometrics: A New Era of Pharmacotherapy and Drug Development in Low- and Middle-Income Countries

**DOI:** 10.1155/2023/3081422

**Published:** 2023-03-07

**Authors:** Muhammad Usman, Sitaram Khadka, Mohammad Saleem, Huma Rasheed, Bimal Kunwar, Moshin Ali

**Affiliations:** ^1^Institute of Pharmaceutical Sciences, University of Veterinary and Animal Sciences, Lahore, Pakistan; ^2^Shree Birendra Hospital, Nepalese Army Institute of Health Sciences, Kathmandu, Nepal; ^3^Punjab University College of Pharmacy, University of the Punjab, Lahore, Pakistan; ^4^Nobel College, Pokhara University, Kathmandu, Nepal; ^5^Faculty of Pharmaceutical Sciences, Govt. College University, Faisalabad, Pakistan

## Abstract

Pharmacotherapy, in many cases, is practiced at a suboptimal level of performance in low- and middle-income countries (LMICs) although stupendous amounts of data are available regularly. The process of drug development is time-consuming, costly, and is also associated with loads of hurdles related to the safety concerns of the compounds. This review was conducted with the objective to emphasize the role of pharmacometrics in pharmacotherapy and the drug development process in LMICs for rational drug therapy. Pharmacometrics is widely applied for the rational clinical pharmacokinetic (PK) practice through the population pharmacokinetic (popPK) modeling and physiologically based pharmacokinetic (PBPK) modeling approach. The scope of pharmacometrics practice is getting wider day by day with the untiring efforts of pharmacometricians. The basis for pharmacometrics analysis is the computer-based modeling and simulation of pharmacokinetics/pharmacodynamics (PK/PD) data supplemented by characterization of important aspects of drug safety and efficacy. Pharmacometrics can be considered an invaluable tool not only for new drug development with maximum safety and efficacy but also for dose optimization in clinical settings. Due to the convenience of using sparse and routine patient data, a significant advantage exists in this regard for LMICs which would otherwise lag behind in clinical trials.

## 1. Introduction

A successful clinical outcome depends on the achievement of five “rights” which include the administration of the right drug to the right patient at the right time in the right dose through the right route of administration. These five rights are included in the clinical decision-making goals of the pharmacist [1]. After the selection of the right drug for a particular condition, the most important feature for a successful therapeutic outcome is the selection of the right dose. The need for dose individualization is necessary for safe and effective drug therapy as overdosing of drugs can cause toxicity, while underdosing may lead not only to therapeutic failure but also to the development of resistance to pathogenic microorganisms in the case of antimicrobials [2]. Variations in the demographics and pathophysiological conditions of the patients, as well as concomitantly administered drugs, can alter the pharmacokinetics (PK) of the drug and hence can affect the amount and rate at which the drug is available at the target site in the body. These variations can be considered in a quantifiable manner through stepwise covariate modeling (SCM) and incorporated in population pharmacokinetic (popPK) models in order to optimize the dose of a drug in a particular patient.

In addition to the different traditional approaches in the application of PK principles for dose optimization according to the clinical status of the patients, there have been different advanced concepts for the rationalization of therapeutic outcomes in clinical pharmacology for clinical PK practice that has added a new facet in clinical drug development and pharmacotherapy process. Pharmacokinetic/pharmacodynamics (PK/PD)-modeling links dose-concentration and concentration-effect relationships, thus easing the depiction and prediction of the time course of drug effects. PK/PD-modeling is a mathematical technique that predicts the changes in drug efficacy over time and drug exposure. Consequently, developments in mathematical modeling, computational power, and convenience of large preclinical and clinical data sets led to the foundation of the quantitative systems pharmacology (QSP) discipline. It is a relatively new field that pools biophysically detailed mechanistic models of physiology with PK/PD to predict systemic effects. QSP has a wide range of applications from creating and investigating new mechanistic hypotheses of a perceived effect, recognizing ideal or substitute targets, attainment of confidence in the rationale of current and/or emergent targets, designing clinical trials, and specifying insight from preclinical to clinical or cross-disease translation to the case of repurposing of the previously developed drug [3].

Pharmacometrics is one of such advanced concepts, which is the science of mathematical modeling and simulation and is defined, according to Barrett et al., as “a science of quantitative models in biology, pharmacology, and disease progression that describes the PK/PD behaviors of drugs with respect to their actions including therapeutic and toxic effects [4].” Thus, pharmacometrics can be considered the amalgam of pharmacology, physiology, pathophysiology, mathematics, statistics, and *in-silico* modeling through computer software programs to meet the regulatory and therapeutic requirements for new drug development and clinical decision-making, respectively [5]. Global utilization and sharing of clinical data based on an understanding of ethnic differences or similarities and appropriate application of new methodology will contribute to more efficient drug development and more scientific decision-making [6]. Clinical pharmacometrics practice has a key role in the optimization of therapeutic outcomes for proper pharmacotherapy practice, where clinical pharmacometricians prospectively apply the models for active therapeutic drug management [7]. Pharmacometrics is a vital tool for the consolidation of a wide range of preclinical and early clinical data through drug exposure disease models in clinical drug development, beyond models to simulate different designs of clinical trials and to decide about the further progression of the process [8]. The practice of pharmacometrics can play an important role to optimize the use of medicines through precise dosing in certain vulnerable populations, such as children, the elderly, and patients with concomitant diseases or comedications in LMICs. However, such practices are being neglected in LMICs due to the lack of experts, intentions related to competency, and endorsement from a regulatory authority.

## 2. Methodology

Literature searches were performed to identify published articles on pharmacometrics in major databases: PubMed, Google Scholar, ScienceDirect, and Cochrane Library. The relevant articles published from January 2000 to February 2022 were studied in detail, and the manuscript was prepared. The experts in the field of pharmacometrics reviewed and finalized this narrative review.

The terms “Pharmacometrics” or “Modeling” and “Simulation” “Population pharmacokinetic study” or “Physiologically based pharmacokinetic study” and “Drug development” or “Drug discovery” or “Pharmacotherapy” or “Clinical decision-making” were the keywords used to search for articles.

## 3. History and Progress of Pharmacometrics

The term “pharmacometrics” was included as a special section in the Journal of Pharmacokinetics and Biopharmaceutics in 1982 [9]; however, its history dates to 1971 and 1976 with the appearance of first citations in articles by Lee [4] that have prognosticated the rays of hope for the development of its practice as an important facet in the area of clinical pharmacology. According to Gobburu, the concept of pharmacometrics began prior to 1960 [5]. In the period of 1950–1970, the pharmacometrics approach was addressed with the development of PK/PD concepts where scientists quantified PK from the lab experiments and developed methods to link PD which was an important tool to develop innovative and advanced concepts for the analysis of data from the clinical trial. The advanced concept of computational skills such as econometric and biometric methods and mixed effect modeling as a headway to quantify observational data patterns has further enhanced the practice in the period of 1970–1980. Lewis Sheiner and Stuart Beal are considered the originators of the scientific discipline of pharmacometrics, and they also developed the NONMEM software system in the 1970s for the popPK study [10]. Likewise, during the period of 1980–1990, the endorsement of pharmacometrics practice by the regulatory authorities made the scope higher. During the same period, in 1989, the United States-Food and Drug Administration (US-FDA) called for pharmacological screening regarding drugs used in the elderly to understand the sources of variability from sparse PK information from the registration trials for the knowledge of the source of variability. The regulatory guidance was issued for the industry for population analysis in 1999, which was an important step in pharmacometrics practice in 1990–2000, and such guidance was also issued for exposure-response relationships in 2003 [5]. Since then, the pharmacometrics practice has had a high-level impact on decisions such as clinical trial design, drug development, approval, and labeling as well as designing drug dosage regimens for the rationalization of pharmacotherapy practice in the clinical settings.

## 4. Organizations and Meetings

A number of organizations are working in different geographical regions and conducting meetings on an annual or occasional basis. These organizations include the Population Approach Group Europe (PAGE), American Society of Pharmacometrics (ASoP) which has evolved into the International Society of Pharmacometrics (ISoP), Population Approach Group Australia and New Zealand (PAGANZ), World Conference of Pharmacometrics (WCoP), Asian Pharmacometrics Conference (ACP), and Asian Pharmacometrics Network (APN). The PAGE started its local meetings during the early 1990s on an annual basis, and the first PAGE meeting was held on June 1992 in Basel, Switzerland [11]. The first meeting of ISoP with the title of the American Conference on Pharmacometrics (ACoP) was held in March 2008 in Tucson, Arizona [12]. The PAGANZ started its annual meeting with the first meeting held in January 1999 in Auckland, New Zealand [13]. The meetings of WCoP were held in September 2012 in Seoul, Korea [14], in August 2016 in Auckland, New Zealand [15], and in April 2020 in Cape Town, South Africa [16]. The one and only ACP was held in Kyoto, Japan, in October 2017 [17]. The Iberoamerican Pharmacometrics Network (RedIF), particularly relevant to LMICs, started its symposium after its establishment in 2017 in Uruguay. The second, third, and fourth RedIF congresses were organized in Mexico in 2018, Cuba in 2019, and Brazil in 2022 [18, 19]. Likewise, the Asian Pharmacometrics Network (APN), which was formed in September 2012 with a conference in South Korea, organized the initial symposium in Thailand in 2019 and the second symposium in India in November 2020 [20].

Such organizations and meetings served as an excellent platform for the exchange of ideas and technology in the form of satellite workshops for the advancement and recognition of practice worldwide [5]. Now, more advanced and technical research is being carried out on building quantitative disease and drug trial models and simulation studies for clinical drug development and pharmacotherapy practice as well. The facility provided to researchers to quantify the completeness of the information made pharmacometrics analysis more effective as compared to conventional hypothesis testing. Pharmacometrics practice has also given a great impact on the concurrence of drugs against bioterrorism and for emergency preparedness as well [5]. The practice of pharmacometrics is still in its infancy in LMICs, though efforts are being made by experts in the said area. With the effort of strong groups of different countries such as RedIF and APN in enhancing pharmacometrics research and innovation within health sciences and promoting educational activities, LMICs are considered an important ground for pharmacometrics practice.

## 5. PK-PD Modeling in Practice

**Figure 1:**
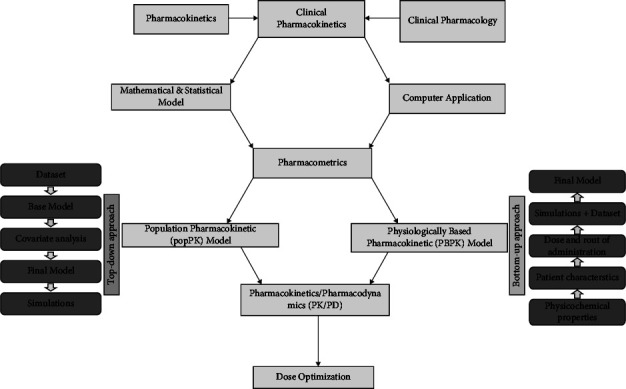
The schematic diagram showing the components of pharmacometrics.

The model is a schematic representation of a complex PK and PD phenomenon that is based on the mathematical relationship and is used to explain and predict the overall behavior of drugs in the body. Modeling is a significant tool in the clinical drug development process and pharmacotherapy practice. Population modeling is a complex process that demands vigorous underlying procedures to ensure clean data, appropriate computing platforms, adequate resources, and effective communication. It is an economic and time-saving practice with the provision of a good framework for the integration of all information collected on new drugs [21]. The developed model can be extended to make it applicable for simulation to solve the queries regarding different study designs. The simulation settings are applied for obtaining an effect of these designs on study outcomes which are further used for subsequent decision-making related to the clinical aspects such as optimization of drug dosage regimen or regulatory affairs such as drug approval [22]. The pharmacokinetic modeling approach for the practice of pharmacometrics is widely applied through the popPK and physiologically based pharmacokinetic (PBPK) modeling approaches (Figure 1).

PBPK and popPK models hold a strong descriptive as well as predictive potential that helps in decision-makingall-around drug development activities and pharmacotherapy approaches in clinical settings. These are complementary and not mutually exclusive techniques in the practice of pharmacokinetics. popPK models can be traditionally considered an empiric model as theoretical features, and even those which are not held up by any specific data but rather by the apprehension of drug characteristics can be commonly included in these models. There is a need for adequate understanding and assessment to confirm the application of these tools like whether to apply one or a hybrid of the two according to the particular queries and the type of data at hand [23]. There are different software packages available for the practice of pharmacometrics, and among them, NONMEM 7.4 by ICON, Phoenix WinNonlin NLME by CERTARA, Simcyp by CERTARA, and Monolix by Lixoft are popular [2, 24, 25]. The nlmixr2 is also common as well as a free and open source [26]. Similarly, PK-Sim and MoBi are maintained by open systems pharmacology and are also standard and free to use [27].

### 5.1. Population Pharmacokinetic (popPK) Modeling

**Figure 2:**
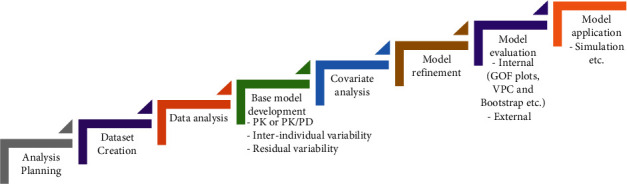
Use of pharmacometrics for dose tailoring. GOF: goodness-of-fit; VPC: visual predictive check.

The variability among individuals after the administration of therapeutically relevant doses is studied in the popPK study where the sources of variability are also investigated that help in dose optimization for the individual patients [2, 28]. A popPK modeling approach is a data-driven approach as the process is started by using the clinical data for base model development, the influence of covariates on PK parameters is evaluated and quantified in the form of mathematical equations, and the magnitude of interindividual as well as intraindividual variability are included in the model [8]. The developed model then can be used to predict a patient-specific model in which simulations of different dosage regimens can be performed for dose tailoring (Figure 2) [22].

**Figure 3:**
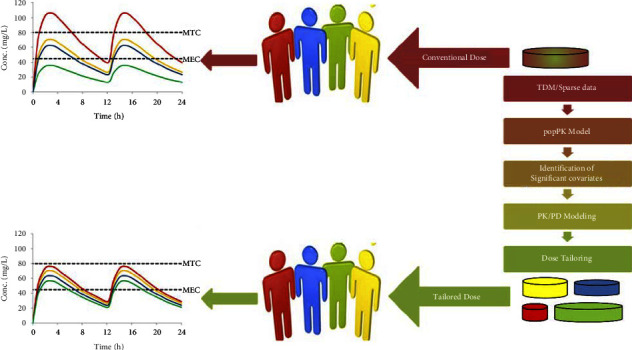
Flow diagram for population pharmacokinetic modeling.

The parameters for the popPK model can be divided into two types, namely, fixed effect parameters and random effect parameters. Fixed effect parameters have a particular value representing clearance and volume of distribution. The random effect parameters include interindividual variability, errors related to the sampling, and bioanalytical techniques [29]. For performing popPK modeling, nonlinear mixed effect models are considered standard. Nonlinear mixed effect models have the unique feature of analyzing sparse data, pooling data from different studies, and dosing simulation in new circumstances. With the development of such a technique, popPK analysis has become enormously convenient not merely for the development of innovative drugs [30] but also for the improvement of already approved drug's therapy and its repurposing practice as well [31]. The popPK analyses are a crucial aspect of almost all drug development programs. Because of the growing emphasis placed on popPK analysis by regulatory authorities and the wealth of information that these analyses provide, it is more important than ever to consider how popPK modeling practice fits into the drug development program. The schematic diagram of the popPK model is shown in Figure 3.

### 5.2. Physiologically Based Pharmacokinetic (PBPK) Modeling

PBPK modeling is a complex, flow-based type of compartment PK model based on the known anatomy, physiology, and blood perfusion to different organs of the species under study. Independent data sources like studies already published and *in vitro* experiments are taken to select the parameter value probability distributions, and the models are assumed validated with the simulated system output adequately akin to a separate data set [8]. PBPK modeling is a theory-driven approach that initiates with what is perceived at the organ or tissue level. The drug movement and disposition in the body are related to the blood flow, concentration of drug in the blood, and a partition coefficient. Such types of models are effective in answering very specific queries; for example, the change in the concentration of a drug in a specific patient population can be speculated by the PBPK model where the molecular signature of the disease has already been included by altering key parameters. Thus, a PBPK model developed for healthy individuals may be modified with respect to the pathophysiological status of the disease. The PBPK model can be extrapolated from one set of patients to other populations and clinical status [32]. These models may incorporate artificial organs as different compartments, and the change in PK of drugs in such scenarios may also be predicted for tailoring of drug dosage regimen [33]. These types of predictions assist in dosing recommendations or impact the design of clinical trials to assess a drug for novel use. Likewise, with the known pharmacokinetic information, drug interactions can be speculated and simulated. PBPK is more precisely described as a specific aspect of QSP that expresses the PD of drugs also in tissues and organs along with elucidating the concentration of drugs in a particular compartment as a function of time.

## 6. Clinical Drug Development and Pharmacotherapy Practice

The clinical drug development process makes a new drug available for the patients' use in the market after the identification of a lead compound through the drug discovery process [34]. This comprises preclinical studies on animals, approval from a regulatory body for an investigational new drug, clinical trials on human volunteers, and again regulatory approval with a new drug application for marketing purposes [35, 36]. The postmarketing phase of the clinical trials continues even after the marketing of the drug for patients' use [37].

The pharmacotherapy practice then makes the proper decision regarding appropriate drug selection with adequate dose and duration of the treatment based on the physiological status and clinical condition of patients. Such a process takes the consideration of risk-benefit judgment and optimizes the dosage regimen that is within the therapeutic range, safe, and effective as well for individual patients [38].

The pharmacometrics techniques can be implemented for the progression of the more efficient process of drug development and rational drug therapy (RDT) practice. The process of drug development is time-consuming as it may take at least 10–12 years to reach the market for use, costly as it takes approximately $2.6 billion investment, and is also associated with loads of hurdles related to safety concerns of the compounds [39]. The opportunity to reach the market eventually often favors very few compounds in the development process and can be withdrawn even after being marketed [40, 41]. Therefore, it is obvious that there must be answers to the few queries that arise in such a process regarding the selection of drug candidates, progression or termination of the project, worthy of the development of a new formulation of improved features for efficacy, adjustment of dosage as well as pharmacokinetic study in a subpopulation, and study of drug-drug interactions to be performed. Application of the PK-PD guided approach as a pharmacometrics principle with the inclusion of modeling and simulation is the best answer in such case, which proves that pharmacometrics has a prime role in the RDT practice through the proper drug development process and pharmacotherapy practice. The FDA Modernization Act of 1997 has boosted it up with the statement that “data from one adequate and well-controlled clinical investigation and confirmatory evidence, obtained prior to or after such investigation, are sufficient to establish effectiveness.” A popPK model established during drug development can provide this confirmatory evidence. The modeling and simulation techniques apply mathematical and statistical models that are, of course, a simplified elaboration of the complex systems under experimentation. The integration of PK&PD principles, which apply PK-PD models to explain the relationship between drug dose, concentration, and pharmacologic response such as surrogate markers, efficacy measures, and adverse drug reaction (ADR) events, is an important process in streamlining and thus making more rational and effective drug development tasks. The design of clinical trials to be undergone in a clinical drug development process comprises a number of specifications with respect to study population, drug dosage, and assessment by geographically spread multidisciplinary development teams. The computer simulation technique based on adequate PK-PD models is the key to assessment to decide on such specifications in an appropriate way. The PBPK models are also being effective for rationalization of pharmacotherapy practice by integration of disease progression and patient compliance. The popPK models also integrate a relationship between different covariates such as body weight, age, sex, race, and parameters of PK-PD that permit assessment and quantification of sources of variability in exposure and response even in sparse sampling conditions in the target population. Stochastic simulation is extensively applied for the assessment of statistical methodology together with the evaluation of bioequivalence parameters. The application of modeling and simulation techniques has also been expanded to speculate outcomes of planned trials in clinical drug development, for which the popPK model together with the random sampling technique is the basis of the approach. For proper simulation to give a real situation, models for disease progression and behavioral features are to be included in the popPK models which help for the evaluation of the outcome of design features for safety as well as efficacy assessment of the drug, thus making effective recognition of statistically valid and practically sensible study designs. The consistent involvement of scientists from different parts of the world specialized in different fields for the practice of pharmacometrics, with no doubt, leads to the production of good quality medicines with maximum efficacy, minimum toxicity profiles, and assures the provision of rational pharmacotherapy practice with optimum drug dosage regimen for the patients [42]. The advantages of pharmacometrics are given in Table 1.

## 7. Need of Pharmacometrics in LMICS

Under the recent World Bank criteria, countries with a gross national income (GNI) per capita up to 12,535$ fall in LMICs [43]. The financial burden, stress, and prolonged hospital stay are treacherous factors for noncompliance in the people of LMICs that lead to therapeutic failure and promote more adverse consequences. The increasing disease burden and difficulty in the availability of medicines and medical services are other gigantic issues in LMICs. The prevalence of chronic and noncommunicable diseases is a common challenge in today's healthcare system in such areas with also a parallel burden of communicable diseases [44]. The healthcare systems in such areas are not able to provide appropriate care due to the lack of comprehensive pharmacotherapy approaches, problems with the availability and affordability of essential medicines, and treatment modalities [45]. The traditional approach of TDM, where peak and/or trough drug concentrations are used for making clinical decisions on dose optimization, is being practiced. In addition to TDM, other approaches such as nomograms, classical noncompartmental equations, or formulae are also not fulfilling the demand for RDT in the growing scenario of complexity in disease and disease progression, comorbidities, different physiological conditions, polypharmacy, genetic polymorphism, complexities in dosage form, and actions. Therefore, the advanced concept in such area for rationalization of therapeutic outcomes is the need of today's healthcare society of the world and pharmacometrics is an appropriate concept in this regard.

Despite the prodigious availability of data on a regular basis in LMICs, the pharmacotherapy practice is mostly at suboptimal levels and so is the case with improvement in drug development. In such cases, pharmacometrics can play a key role in effectively improving drug development and pharmacotherapy practice with the help of its principles and models. Pharmacometrics has a broad spectrum from basic research of clinical trials to the more complex and advanced research on drug discovery and development to pharmacotherapy practice such as the treatment approach of diseases and optimal drug use in the clinical setting of patient care. The practice of drug utilization review (DUR), generic drug concept, substitution concept, and managed care organization bidding are increasing insistence for the drug development process in the pharmaceutical industry for the production and supply of highly safe and effective as well as affordable therapeutic agents. The US-FDA has also highlighted the urgent need for superior product development science in the white paper “*challenge and opportunity on the critical path to new products*” in 2004 regarding the question of cost-push inflation and inadequate application of scientific tools for the safety and effectiveness of products leading to stagnancy in clinical drug development [46]. Pharmacometrics has played an important role in the decision for pivotal US-FDA approval of Toradol (Ketorolac), Natrecor (Nesiritide), Remifentanil, and Neurontin (Gabapentin) and has been the prime factor to impact on such concerns of FDA tools for assessing safety, demonstrating medical utility, and characterization and manufacturing [5, 6]. The division of pharmacometrics has become an important part of regulatory affairs for decision-making and establishing product labels in new drug applications (NDAs) in US-FDA [5]. According to a survey of 198 applications for drug approval and labeling during the period of 2000 to 2008, pharmacometrics was found to play a role in the decision-making of 60% of applications [47]. Moreover, it has also played its role in the inclusion of two-step dosing regimens of Busulfan for the product label in the pediatric section and revision of the language of the product in terms of dose recommendation of pasireotide injection used in Cushing's disease [22]. Scientific approaches of pharmacometrics have tremendous effects on clinical drug development and optimization of the dosing strategy, which involves highly effective computational speed, novel models, stochastic simulation models, real-time data collection, and novel biomarkers. The important pharmacokinetic covariates such as patients' age, size, gender, renal profile, and hepatic functions are essential parameters but not studied at an adequate level for optimizing dosing strategies, and these are linked to the model development in pharmacometrics for clinical drug development and pharmacotherapy practice for rationalization of the therapeutic outcome [6]. Pharmacometrics is now being practiced throughout the phases of the process of drug discovery and development together with regulatory approval to marketing the product. Therefore, it has an assimilating application in the transformation to model-based development [48].

In the context of an emergency such as the COVID-19 pandemic, where proven treatment option is still a far cry, repurposing of previously discovered drugs became a popular and effective approach [49]. Some of the drugs used globally are Hydroxychloroquine, Azithromycin, Remdesivir, Favipiravir, Dexamethasone, Ivermectin, and Tocilizumab [50]. However, the safety profile remains an issue; particularly, the evidence of benefit is highly controversial in the case of Hydroxychloroquine, Ivermectin, and Dexamethasone. Different episodes of cardiac adverse events and neurological toxicities were associated with the use of Hydroxychloroquine with or without Azithromycin and Ivermectin, respectively [49, 51–57]. The increased mortality risk with the use of corticosteroids was also reported [58]. Clinical improvements following the treatment with Favipiravir and Remdesivir have been documented though proofs of inefficiency in viral clearance have also been reported [53, 59, 60]. The critical analysis of the clinicians considering the risk-benefit judgment according to the clinical status of patients is a key to the use of these agents in such a global health crisis [51, 58–60]. In such a scenario, pharmacometrics plays an essential role that optimizes drug dosage regimens for beneficial outcomes [31].

Though the need for pharmacometrics practice is important globally as it has not been given much more emphasis in most parts of the world, LMICs have to adopt such practice immediately. The pharmacometrics practice has not taken a quantum jump in LMICs due to the availability of very few pharmacometricians in number or virtually nonexistent in most of the parts. However, the LMICs can be a venue for practice for pharmacometricians to develop relevant local solutions to global healthcare problems [61]. Thus, pharmacometrics is a suitable and readily applicable concept in healthcare delivery in LMICs for developing safe, effective, accessible, and affordable products with maximum efficacy and minimum toxicity in different age groups and clinical status of patients [62].

## 8. Challenges and the Way Forward

Pharmacometrics is now widened to a much extent as compared to the initial phase of its development and practice with the untiring efforts of a multidisciplinary team consisting of pharmacometricians, academic researchers, drug development scientists, regulatory experts, and other related professionals of the healthcare field. To upgrade productivity to the current scope and to bring advances in practices for broadening the future scope of pharmacometrics practice are common strategic goals as well as a challenge in this field. The inadequate number of perfectly trained scientists, reluctance in sharing knowledge and information in some cases, and reluctance to recognition of applications can be regarded as a weakness in practice. Though the practice has great potential and is up to a certain level in developed countries, it is still neglected in LMICs despite the effort of some experts. Therefore, there is a substantial need for such practice in LMICs for safe and effective drug therapy as the financial burden, and the literacy rate, multicomorbidities, resistance to drug therapy, and poor compliance are challenging factors in such areas. Such factors along with the genetic makeup of individuals as well as physiological status are actually challenging the world's health scenario, and there is a need for innovation and challenges in dosage form design and hence drug development and rational practice to cover such conditions. This will definitely make the area of scope of pharmacometrics wider, more interesting, and more challenging as well. Growing expectations from patients' sites, increase in niche products, global drug development, and more efficient safety assessment are external factors as well as potentials that drive the opportunities and expand the scope of practice of developing safe and effective drugs for rational pharmacotherapy practice [5]. Appropriate formulation with proper dosage regimen according to the specific patient population and clinical status is needed to be developed and practiced consistently at an affordable price at all levels in LMICs to make the healthcare system effective.

The systematic implementation of pharmacometrics in drug discovery and development has a prospective role in significantly enhancing medical breakthroughs and making advanced treatment options available. Such challenges have focused on the requirement of proper education and training at the academic as well as at the professional level, proactive and effective planning, dynamic access to multisource data, an amalgamation of quantitative knowledge, inter/intraprofessional collaboration, and effectual dissemination of innovative and impactful application of pharmacometrics [63]. The development of a model and building relationships between different fundamental PK parameters and patient demographics help the experts develop an individualized dosage regimen for RDT related to the clinical status of patients. Pharmacometrics analysis has better insight as compared with other available techniques to solve the query related to the safety, efficacy, and individualized drug dosage regimen of drugs in related clinical status [2].

The integration, innovation, and impact can be considered as the three overarching themes that helped the development of pharmacometrics to come up to this stage and have the potential to expand the field even more with the innovation and advancement in practice on drug discovery and development, research, regulatory approval, and utilization of new medicines for RDT [64]. Applications of pharmacometrics principles help design efficient studies and interpret their results in the context of all available data and knowledge to enable effective decision-making for the clinical drug development processes and pharmacotherapy practices [65]. The pharmacometrics practice field has come a long way concerning its size and achievements with the hard work of pharmacometricians. The supply is drawing high demand for such practice effectively despite the complexity and quantity of demand [5]. The existence of a vacuum between academia, clinical setups, and the pharmaceutical industry leading to the poor recognition of this practice must be removed to make it highly effective.

For the further progression of the practice of pharmacometrics, regulatory bodies should give emphasis on its implications as well as provide education and training to every related field ranging from academics to the pharmaceutical industry. In academics, implementing a syllabus to study theory and research work; in a clinical setting, implementing the principle of pharmacometrics for the pharmacological treatment of different types of diseases with the innovative approach and tailoring of dosage regimen for rationalization of the therapeutic outcome; in the pharmaceutical industry, implementing the principles of pharmacometrics for the development of a new and advanced type of drugs and dosage form with effective mechanism and approach which demonstrates minimum toxicity and maximum safety can be a significant tool in health science. Such steps may lead the LMICs toward a better healthcare system and facility.

## 9. Conclusion

The foundation for pharmacometrics analysis is the *in-silico* modeling and simulation of pharmacokinetics/pharmacodynamics (PK/PD) data complemented by a description of significant features of the safety and efficacy of the therapeutic agent. As an extensively applied tool for the clinical pharmacokinetics practice through the population pharmacokinetic modeling and physiologically based pharmacokinetic modeling approach, the computer-based modeling and simulation technique of pharmacometrics is a significant approach in proficient clinical drug development and pharmacotherapy practice. The healthcare system of the world, especially LMICs, which lag behind in advanced technology, innovation, and clinical trials, can get the benefit of using sparse and routine patient data for proper implementation of such practice that ensures rationalization of therapeutic outcomes. The support of the regulatory body and consistency of healthcare professionals in such areas are crucial to broadening its scope and healthcare facility.

## Figures and Tables

**Table 1:** Advantages of pharmacometrics.

Subjects	Areas	Particulars
Pharmacometrics	Clinical drug development	To inform and optimize dose selection
		To inform route of administration, bioequivalence, and formulation decisions
		To help determine the route of administration of drugs
		To quantify exposure-response relationships for efficacy and safety and subsequent clinical utility
		To help repurposing of the drug
	Pharmacotherapy practice	To optimize the drug dosage regimen in special patient populations such as pediatrics, geriatrics, pregnancy, lactating women, genetically different patient populations, and patients with renal and liver impairment
		To optimize and individualize dosing to ensure optimal efficacy and safety

## Data Availability

No data were used to support the findings of this study.
